# Genome-Scale Metabolic Network Validation of *Shewanella oneidensis* Using Transposon Insertion Frequency Analysis

**DOI:** 10.1371/journal.pcbi.1003848

**Published:** 2014-09-18

**Authors:** Hong Yang, Elias W. Krumholz, Evan D. Brutinel, Nagendra P. Palani, Michael J. Sadowsky, Andrew M. Odlyzko, Jeffrey A. Gralnick, Igor G. L. Libourel

**Affiliations:** 1 Department of Plant Biology, University of Minnesota, St. Paul, Minnesota, United States of America; 2 BioTechnology Institute, University of Minnesota, St. Paul, Minnesota, United States of America; 3 Department of Microbiology, University of Minnesota, Minneapolis, Minnesota, United States of America; 4 Department of Soil, Water, and Climate, University of Minnesota, St. Paul, Minnesota, United States of America; 5 School of Mathematics, University of Minnesota, Minneapolis, Minnesota, United States of America; The Pennsylvania State University, United States of America

## Abstract

Transposon mutagenesis, in combination with parallel sequencing, is becoming a powerful tool for *en-masse* mutant analysis. A probability generating function was used to explain observed mini*Himar* transposon insertion patterns, and gene essentiality calls were made by transposon insertion frequency analysis (TIFA). TIFA incorporated the observed genome and sequence motif bias of the mini*Himar* transposon. The gene essentiality calls were compared to: 1) previous genome-wide direct gene-essentiality assignments; and, 2) flux balance analysis (FBA) predictions from an existing genome-scale metabolic model of *Shewanella oneidensis* MR-1. A three-way comparison between FBA, TIFA, and the direct essentiality calls was made to validate the TIFA approach. The refinement in the interpretation of observed transposon insertions demonstrated that genes without insertions are not necessarily *essential*, and that genes that contain insertions are not always *nonessential*. The TIFA calls were in reasonable agreement with direct essentiality calls for *S. oneidensis*, but agreed more closely with *E. coli* essentiality calls for orthologs. The TIFA gene essentiality calls were in good agreement with the MR-1 FBA essentiality predictions, and the agreement between TIFA and FBA predictions was substantially better than between the FBA and the direct gene essentiality predictions.

## Author Summary

Metabolic modeling techniques play a central role in rational design of industrial strains, personalized medicine, and automated network reconstruction. However, due to the large size of models, very few have been comprehensively tested using single gene knockout mutants for every gene in the model. Such a genetic test could evaluate whether genes that for a given condition are predicted to be essential by a model, are indeed essential in reality (and vice versa). We developed a new probability-based technology that identifies the essentiality of genes from observed transposon insertion data. This data was acquired by pooling tens of thousands of transposon mutants, and localizing the insertion locations all at once by using massive parallel sequencing. We utilized this gene essentiality data for the genome-scale genetic validation of a metabolic model. For instance: our work identified nonessential genes that were predicted to be essential for growth by an existing metabolic model of *Shewanella oneidensis*, highlighting incomplete areas within this metabolic model.

## Introduction

Transposon mutant analysis has been extensively used to generate genome-wide mutant libraries and to define gene essentiality [1], [2]. With the introduction of parallel sequencing, transposon-based methods have developed into phenotype information gathering tools, rather than forward genetic screens with the aim to isolate individual mutant strains. Tn-seq, and closely related methods such as Bar-seq or DNA shearing [3], investigate mutant fitness at a genomic scale by counting the abundance of a mutant-specific DNA sequence before and after a short competitive growth period [4], [5]. In Bar-seq the unique piece of DNA is located between known flanking sequences and can be sequenced directly [6]. In Tn-seq, a type IIS restriction enzyme that cuts outside its recognition sequence is used to extract transposons from the mutant genomes, including a flanking sequence (17 bp for mini*Himar*) that is used to map the location of the transposon insertion.

Transposon insertion sequencing has been used to identify essential genes in an increasing number of microorganisms from a wide range of ecological niches [7]. Of particular significance is the application of Tn-seq to infectious agents in order to identify essential genes that could serve as targets for therapy [8]–[12]. However, fitness due to disruption of coding sequences is not the only type of data that has been obtained from this method. When transposon mutant libraries were generated to genome-saturating conditions, the essentiality related to disruption of non-coding regions was identified [13], [14], facilitating the identification of non-coding regulatory elements. In *S. oneidensis*, himar and Tn5 transposons have been used to identify a number of mutants and elucidate cellular physiology [15]–[17]. Barcoded genome-wide mutants of *S. oneidensis* have been created with the himar transposon and their individual fitness evaluated in a large number of growth conditions using microarrays [18]. The creation of tagged transposon mutant libraries has also enabled systems-level analyses of *S. oneidensis*, such as mass-spectrometry based metabolite profiling of mutants [19] and computational inference of gene regulatory networks based on fitness data [20].

Transposon mutagenesis-based gene essentiality measurements are exceptionally informative for the validation of genome-wide modeling techniques. The genome-wide scale and low-cost nature of disposable single gene knockout libraries provide powerful datasets to evaluate the performance of genome-scale network reconstructions [21], as well as to enrich genomic information currently used for automated network reconstructions [22]–[24].

The presented transposon insertion frequency analysis (TIFA) improves on the more direct interpretation in which presence of an insertion in the gene core is interpreted as sufficient evidence of nonessentiality, and absence of insertions is interpreted as essentiality [18], [25]. The need for a more sophisticated approach has been recognized by others, with recent estimations of gene essentiality using hidden Markov models (HMM) [26], [27]. TIFA distinguishes itself from an HMM approach by considering insertional biases in sequence preference and genomic location for each insertion. The insertional biases of the mini*Himar* transposon in *S. oneidensis* were investigated using an existing dataset [28]. TIFA determines the likelihood of the number of experimentally observed insertions in each gene and utilizes a probability generating function that accounts for observed insertional biases of the mini*Himar* transposon. The thus determined gene essentiality for growth on *Shewanella* Basal Medium (SBM) under aerobic conditions was used to investigate the stoichiometric and thermodynamic constraints on the existing MR-1 metabolic model [29], simulating aerobic growth on SBM.

## Results

### mini*Himar* transposon insertions frequencies are location and sequence biased

**Figure 1:**
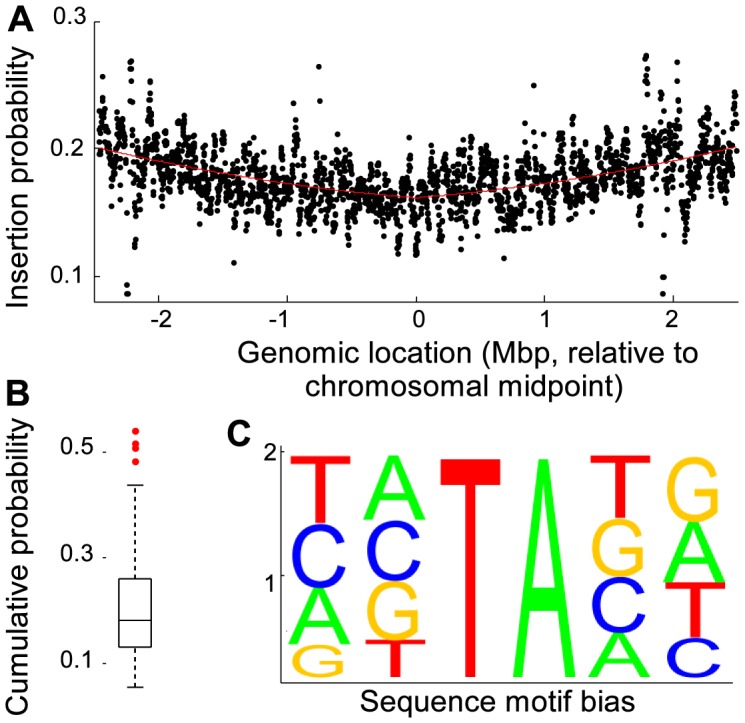
Evaluation of transposon insertion bias. (A) Scatter plot of the genome location dependent insertion probability. Each data point represents a genomic window of 20,000 nucleotides, which was shifted by 2,000 nucleotides for each consecutive evaluation. The insertion probability gradually decreased from the origin of replication. The solid line is a best fit of ax^2^+b|x|+c, where a, b and c are equal to 0.0032, 0.0081 and 0.1615, respectively, and x is the location in the genome. (B) Boxplot of flanking sequence dependent insertion probability distribution showing that several motifs contained significantly more insertions than others. (C) Relative occurrence of nucleotides in the two flanking positions of inserted TA sites.

The mini*Himar* transposon has a strong preference to insert inside a TA sequence [30]. The flanking sequences of the chromosomal transposon insertion library confirmed this preference, with just over 95% of the mutations located inside a TA sequence. We discarded the remaining ∼5% of insertions and investigated the TA inserted transposons in more detail (Fig. 1). A potential bias in the *chromosomal* insertion position was investigated by plotting the insertion frequency as a function of chromosomal location (Fig. 1A). Because the *exact* same insertion may have occurred in several independent colonies, the number of insertions was estimated from the number of TA locations that had *not* been inserted (Material and Methods). The locational bias was quantified by fitting an absolute value second order polynomial through the observed frequency data (Fig. 1A). The insertion frequency showed an approximate 25% symmetrical bias towards the origin of replication compared to the midpoint of the chromosome. Presumably, this bias is the result of the presence of multiple partial copies of an actively replicating circular chromosome [31] near the replication fork, leading to a higher physical copy number of genes closer to the origin of replication.

The effect of the two flanking nucleotides on either side of the target dinucleotide was investigated in detail using genes that contained no fewer insertions (*p*>0.1) than expected from the binomial distribution prediction (Fig. 1B). The dataset was subdivided into 136 sections, each section corresponding to a unique combination of the two flanking nucleotides prior and the two nucleotides following a TA location. The two complementary strands of the genome result in two possible sequence orientations, with each sequence on the plus strand matching a complementary sequence on the minus strand. Sixteen sequences are palindromic, resulting in a total of (256-16)/2+16 = 136 unique sequences. For each of the 136 unique sequence combinations, the occurrences and insertion events were determined as before. The insertion probability was sequence dependent, with 3% of the probabilities differing significantly from the mean probability (Fig. 1B).

To determine if the flanking nucleotides affected the insertion probability independently, we assumed that the number of insertions for each flanking sequence was the product of the independent contributions of the nucleotides. Thus, each of the 136 determined insertion frequencies was given by 
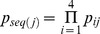
, where 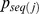 is the observed insertion probability for a given flanking nucleotide combination *j*, and *p_ij_* are the contributions of the individual nucleotides in the combination. Using weights that were inversely proportional to the variance for each sequence insertion probability 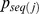, nonlinear χ^2^-fitting was used to calculate the parameter *p_ij_*. Assuming multinomial variance, the observed probabilities differed significantly from a linear model (χ^2^ test, *p*<0.01), indicating that the contribution of the flanking nucleotides on the insertion probability were not independent. Fig. 1C shows the linear approximation of the nucleotide contributions, and although the linear approximation indicated that the TATATG sequence had the highest insertion probability, the independent contributions underestimated the insertion probability for this sequence by 30%. In addition, there were three sequences for which an even higher experimental insertion probability than the experimental value for the TATATG was found. Visual inspection of the experimental sequence probabilities suggested that the preferred consensus sequence was TATAxA. Just TA enrichment alone was not sufficient, exemplified by the barely average insertion probability associated with AATATT. The highest experimental insertion probability was associated with TATATA (*p* = 0.53), and the smallest probability was associated with GTTAAC (*p* = 0.057), indicating an approximate tenfold spread in insertion preference.

### Essential gene calls

**Figure 2:**
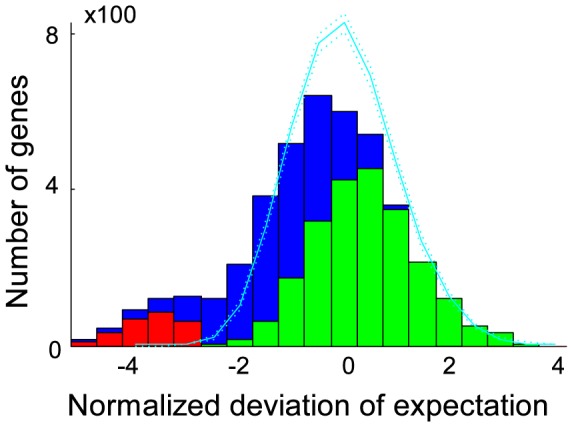
Observed and Monte Carlo simulated transposon insertions. The normalized deviation of expectation of insertions are shown for TIFA essential (red), nonessential (green) and unknown (blue) genes. The solid line represents the average outcome of 1000 Monte Carlo simulations, flanked by one standard deviation (dotted lines).

**Figure 3:**
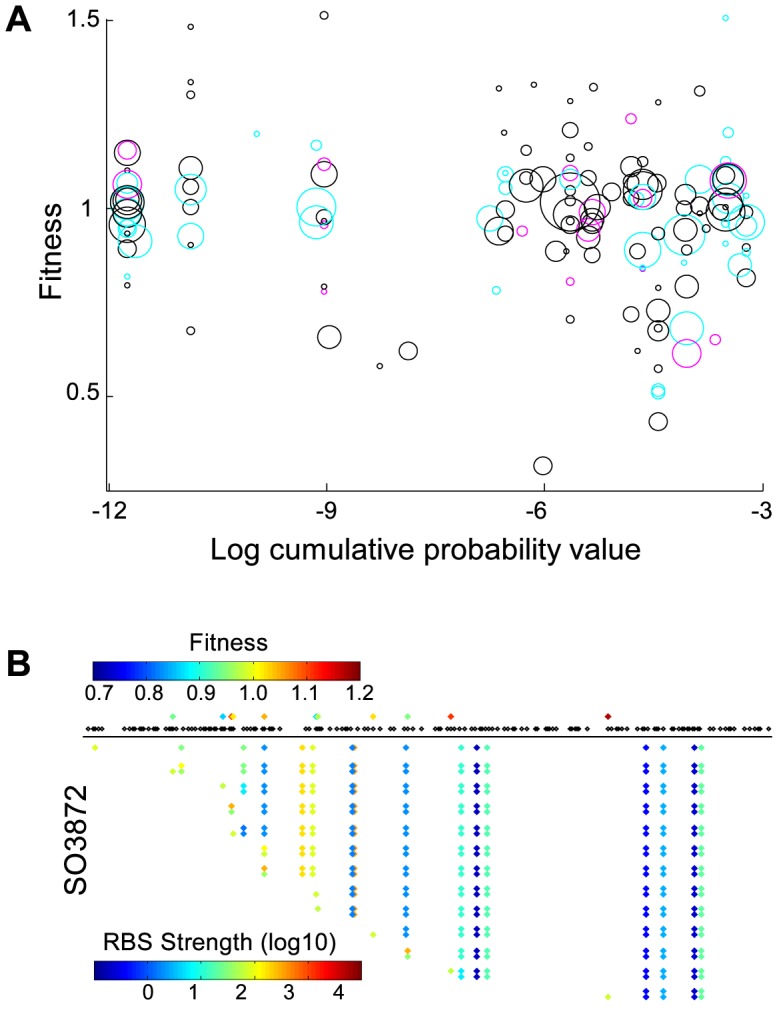
(A) 50 out of 273 essential genes were associated with a fitness value. The circumference of the circles represents the logarithmic read counts of each insertion, and the color of the circles represents the gene location of the insertion: first 10% (magenta), last 10% (cyan), and middle 80% (black). 22 essential genes contained ≥2 insertions in the middle. The spread in fitness and read counts was very large, suggesting different causes for the existence of insertions in essential genes. (B) Transposon insertion locations, fitnesses, and projected ribosome binding site (RBS) strengths associated with intra-gene start codons. Black dots show all TA locations, and top row diamonds show the observed insertions with color coded fitness. The bottom diamonds represent alternative start codons in each gene. The color scales logarithmically with the associated RBS strengths. Each couple of rows represents the RBS strengths of all intra-gene start codons for both possible insertion orientations for each mutant, with the first row representing no insertion.

Transposon mutagenesis results in the random disruption of genes, often reducing or eliminating gene function. In principle, transposon mutagenesis therefore reports on gene essentiality. The essentiality of a gene can be investigated by comparing the number of observed mutations in a gene to the number of expected insertions. A transposon probability model was formulated that accounted for the observed sequence and location specific biases. For each gene, the expected number of insertions was estimated using a probability generating function with the sequence specific probabilities that were weighted by the genome locational specific bias. Using this probability model, the number of expected insertions was calculated for each gene and compared to the observed number of insertions (Fig. 2). Genes were called essential if the combined probability of finding as few as, or fewer insertions than observed, was less than one over the number of genes in the dataset i.e. we accepted one false positive in our essential gene selection. To establish the exact cutoff value, the marginal probabilities for each nonessential gene to be found essential by chance for a given cutoff value were summed, and the cutoff value was adjusted until the marginal probabilities summed to exactly one. Monte Carlo sampling was used to confirm this result, and the same cutoff value was retrieved (Text S1). The transposon insertion distribution was visualized as a histogram of the difference between the expected and observed number of insertions, scaled by the standard deviation calculated on the probability generating function. Monte Carlo sampling generated a very similar distribution (Fig. 2). The right hand side of this distribution is of particular interest, as an elongated tail in the observations could suggest genes with significantly more insertions than expected. Only three such genes were observed (SO3264, SO4100, SO4785), indicating that TIFA formed a good description of the observed transposon insertion behavior. The poorer fit on the left hand side of the distribution could be the result of mutants with a lower than wild type fitness. Clones with a reduced fitness are more likely to escape detection due to their lower absolute abundance, yielding fewer observed insertions in nonessential genes that show reduced fitness upon deletion. Some genes with slow growing mutant phenotypes could have been called essential as a result. For 50 of the 273 identified essential genes a fitness value larger than zero was observed. To investigate if mutants with insertions in genes that were called essential grew more slowly than other clones, the nonessentiality probability was plotted against fitness (Fig. 3A). Although the average fitness of clones with insertions in essential genes was lower than fitness of clones with insertions in nonessential genes, the spread was very large, indicating that slow growth was an insufficient explanation for all detected insertions in essential genes. The insertion locations were plotted together with the positions of conserved domains and, to investigate the potential for reinitiation of transcription and translation downstream of the transposon, estimates of the strength of ribosomal binding sites associated with alternative start codons inside the genes (Figure S1).

### Nonessential gene calls

Following identification of essential genes with a nonessential gene insertion model, the nonessential genes were identified with an *essential* gene insertion model. The insertion frequency in essential genes was approximated by multiplying the TA location-specific insertion probabilities with the ratio of the observed insertions in essential genes by the expected number of insertions for nonessential genes. The expected number of insertions for each gene was calculated using this essential gene model, and each gene that contained significantly *more* insertions than expected, was called nonessential. Following the earlier logic, a cutoff value was used that allowed for a single false-positive nonessential gene identification. This method identified 2,216 genes as nonessential. No essentiality call could be made for 1,722 genes, and three genes (SO2148, SO3175 and SO3872) were identified as both essential and nonessential. Hence, the number of insertions in these three genes was significantly fewer than could be expected for nonessential genes, yet significantly more than could be expected for essential genes. For example, closer inspection of SO3872 revealed that insertions were concentrated in the second quartile and most showed a reduced fitness (Fig. 3B). Two additional insertions in the latter half of the gene showed high fitness, and no other insertions were present. There was very good sequence support for the gene assignment based on the sequence alignment with the arylsulfate sulfo-transferase pfam PF05935 [32]. Conceivably, only the beginning and second half of the gene were essential for the production of a functional protein, and start codons around the midpoint of the gene with associated ribosomal binding sites of moderate projected strength [33], suggested that translation may be reinitiated within the gene resulting in separate expression of the second half of the gene (Fig. 3B). More generally, essential genes could have nonessential regions such as a regulatory site which may be highly inserted, and be adjacent to uninserted regions necessary for the essential gene role, which could cause a dual essential-nonessential identification. Gene essentiality calls that were performed in previous work on the bases of absence or presence of insertions [18] were compared to TIFA calls in detail (Table 1 and Dataset S1). Seventy eight essential gene calls and 1,958 nonessential gene calls were in agreement. Eighty one genes that were previously identified as nonessential (including two orthologs to essential genes in *E. coli*) were identified as essential by TIFA, 36 of which contained no insertions in our dataset. Fifty one of the genes previously identified as essential were identified as nonessential by TIFA, and contained an average of 10 insertions per gene.

**Table 1:** Comparison of gene essentiality calls between TIFA and DEC.

DEC	1Expected essential	2New essential	3Dispensable	4Surprise dispensable	5Dispensable unclear in *E.coli*	Unknown	Total
**TIFA**							
Essential	66 (81)	12 (6)	79 (23)	2 (0)	0 (0)	114 (10)	273 (120)
Nonessential	34	17	1895	6	57	207	2216
Unknown	155	31	881	6	31	501	1605
Total	336	66	2878	14	88	832	4214

The six essentiality classes (columns) that were previously defined [18] for the direct essentiality calls (DECs), were cross compared to the three TIFA essentiality classes (rows). Columns (1,2) represent DEC essential gene calls; columns (3,4,5) DEC nonessential gene calls. The excluded TIFA essential gene calls that could be explained alternatively by operon polar effects are listed between brackets.

1 Essential orthologs;

2 essential gene calls with conflicting or absent corroborating evidence from orthologs,

3 nonessential orthologs;

4 nonessential gene calls with conflicting evidence from orthologs.

5 nonessential gene calls without corroborating evidence from *E. coli* orthologs.

A detailed description of the six DEC categories is given in Text S1 of reference [18].

### Essential genes are inserted more frequently at the very end of a gene

**Figure 4:**
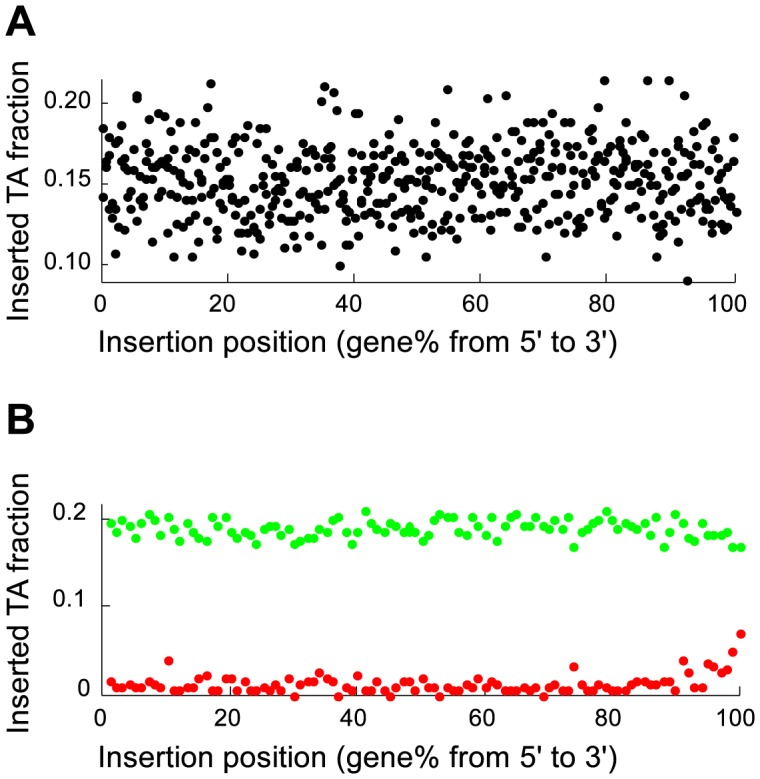
Insertion frequency within genes. Genes were evaluated in 0.2% gene increments. No insertion preference was observed in the complete gene population (A). The same analysis was performed (1% increments) after essential (red) and nonessential (green) gene identification (B). The last ∼2% at the 3′ end of essential genes were inserted more frequently.

The number of TA sequences and the number of detected insertions were tallied for each gene percentile to investigate if the positions of insertions inside (essential) genes were biased (Fig. 4). No percentile showed a difference in relative insertion frequency prior to identification of essential genes (Fig. 4A). Analysis of just the nonessential and essential genes revealed a substantial increase of insertions in the last 2–3% of essential genes. Essential genes were almost twice as likely to contain insertions in the last 2% nucleotides, but insertion frequencies were still only half of the frequencies observed in nonessential genes (Fig. 4B). Nonetheless, the increased insertion frequency at the very end of essential genes provides quantitative support for the omission of insertion data from the last 2% of genes. Previously, much larger areas of genes were excluded from insertion analysis, arguing that insertions in the distal parts of genes may not be effective in eliminating gene function [4]. No relative increase in insertion frequency was observed at the beginning of genes.

### Validation of the genome scale MR-1 model essentiality predictions

The ability to identify essential and nonessential genes with TIFA was utilized to validate essentiality predictions of an existing genome-scale metabolic model of *S. oneidensis* MR-1 that had been manually curated previously [29]. The MR-1 model includes 774 reactions, 783 gene and 634 unique metabolites. Flux balance analysis (FBA) was used to infer gene essentiality for genes that were present in the MR-1. The transposon mutants were selected under aerobic conditions on SB media, and FBA predicted 209 essential genes and 574 nonessential genes in the MR-1 model for these conditions. The annotations of thirteen genes in the MR-1 model were obsolete, which reduced the comparison of FBA to TIFA predictions to 770 genes. TIFA was able to determine essentiality for 481 genes (62%) of this set (Table 2) providing a comprehensive evaluation of the network essentiality predictions.

**Table 2:** Comparison of TIFA gene essentiality and FBA predictions.

	TIFA essential			TIFA nonessential		
	FBA-E	FBA-N	% True	FBA-N	FBA-E	% True
Original MR-1 model	57	18	76	374	32	92
All metabolites can leave model	56	19	75	374	32	92
No thermodynamic constraints	33	42	44	384	22	95
Metabolites can leave, no thermodynamic constrains	33	42	44	384	22	95

TIFA essentiality calls compared to FBA predictions for the MR-1 model: using the original model (row 1); after removal of stoichiometric constraints on endpoint metabolites (row 2); after removal of all thermodynamic constraints (row 3); and following the removal of the stoichiometric and thermodynamic constraints (row 4). FBA-E: FBA essential gene predictions; FBA-N: FBA nonessential gene predictions.

**Figure 5:**
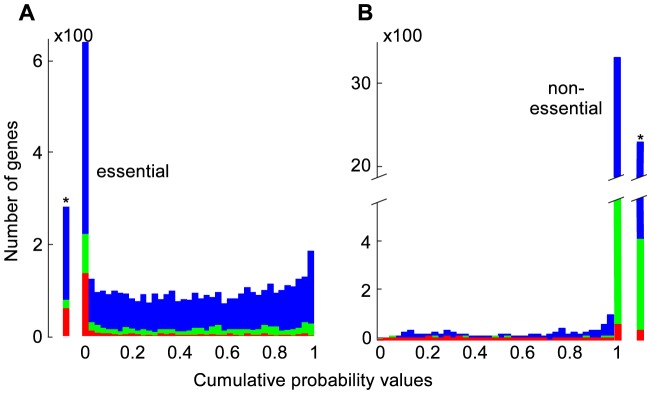
Comparison of gene essentiality between TIFA calls and FBA predictions. Genes were grouped in 40 bins based on their cumulative probability values. The blue bars represent the genes not present in the MR-1 model. The red and green bars represent the FBA essential and FBA nonessential genes. Genes with a lower cumulative probability value are more likely to be essential, and genes with a higher cumulative probability value are more likely to be nonessential. The leftmost bar in (A) shows the number of genes with a cumulative probability value of less than the cut-off value (TIFA essential, allowing for 1 false positive). The rightmost bar in (B) shows the number of genes with a cumulative probability of more than the nonessential cut-off value (TIFA nonessential, allowing for 1 false positive).

Of the 273 identified essential genes identified by TIFA, 75 were present in the MR-1 model. Fifty seven of the included 75 genes were correctly predicted essential, and 374 of the 406 FBA-nonessential genes that were present in the MR-1 model and were identified as nonessential by TIFA (Fig. 5). FBA essentiality predictions were insensitive to the 1% biomass production cutoff, with 1% growth resulting in the same knockout predictions as no growth (Text S1). The TIFA essentiality calls were fairly sensitive to the essentiality cutoff. If for instance the lower 2.5% likelihood of gene *nonessentiality* had been used as essential gene cutoff instead (Fig. 5a), twice as many genes (623) would have been identified as essential suggesting that only half of the essential genes were called. However, the number of false positive would have been much larger (54 genes, calculated from marginal probabilities), and the agreement between FBA and TIFA prediction was indeed substantially better when using the stringent cutoff (Fig. 5a).

### Genes falsely predicted nonessential

False nonessential FBA predictions could be caused by an array of pleiotropic effects, such as the built-up of a toxic metabolite resulting from the removal of a downstream reaction in a pathway. More directly, false nonessential predictions may be caused by a combination of incorrect gene-reaction association, lax thermodynamic constraints, and over-inclusion of metabolic reactions. Such an overly inclusive metabolic network could also arise as a result of the absence of, or an incomplete gene regulatory layer. Blocked reactions, which cannot carry flux under any circumstances, point to the most easily interpretable shortcomings of the network. Eleven of the 18 FBA-nonessential, TIFA essential genes were associated with such blocked reactions (Text S1). These reactions were evidently also not required for biomass formation by the model, indicating that the biomass equation was not sufficiently inclusive to test all essential genes, or in reality unused reactions provided an alternative route to essential biomass. In addition, because the reactions were blocked, TIFA suggested that the MR-1 model requires modifications to unblock these TIFA essential reactions. In the case of aconitase (E.C. 4.2.1.3), the discrepancy between the MR-1 model and the TIFA essentiality data highlighted the need for a comprehensive gene expression and protein activity regulation simulation in metabolic models. In the MR-1 model, *acnB* (SO0432) and *acnD* (SO0343) were independently assigned to the aconitase reaction (OR relationship), resulting in a nonessential prediction for *acnB*. However, an *acnB* deletion strain was unable to grow in the presence of oxygen [28], which was consistent with transposon insertion data.

### Genes falsely predicted essential

Genes that were falsely predicted essential by FBA could be interpreted as resulting from missing reaction in the network, insufficiently permissive thermodynamic constraints, errors in gene-to-reaction relationships, an overly inclusive (essential) biomass equation, or an incorrect regulatory layer. However, pleiotropic effect cannot explain these discrepancies. Of the 2216 TIFA identified nonessential genes, 374 genes were correctly predicted to be nonessential and only 32 were incorrectly predicted essential by FBA (Fig. 5). Of the incorrectly essential predictions, SO1498 and SO3745 would have been correctly predicted nonessential if the two biomass components lipopolysaccharide (LPS) and glycogen were not included in the biomass equation. LPS may indeed not be required for growth [34]. The transporter for ammonia, *amtB* (SO0760) may only become essential at very low ammonia concentrations [35]. A detailed comparison between TIFA and FBA essentiality is shown in Text S1.

### Network constraints and scale

The observed discrepancies between TIFA and the model predictions were further investigated by: 1) allowing metabolites to freely leave the network, and 2) by removing the thermodynamic constrains from the model. Lifting of stoichiometric constraints on endpoint metabolites resulted in a marginal deterioration of essential gene predictions, and no change in nonessential gene predictions (Table 2), suggesting that blockage of reactions due to stoichiometric constraints on endpoint metabolites was not important in FBA gene essentiality predictions for the MR-1 model. Removal of the thermodynamic constraints resulted in a substantial deterioration of essential gene predictions and a very marginal improvement of nonessential gene predictions (Table 2), confirming the importance of correct thermodynamic constraints in gene essentiality predictions. In comparison to a model of central metabolism of *S. oneidensis* MR-1 [36] that was formulated for elementary mode analysis, the much larger scale MR-1 model improved gene essentiality predictions substantially. Only one gene (SO3547) of the previously eight genes falsely predicted essential (SO0424, SO0323, SO0538, SO1926, SO2629, SO3547, SO0274, and SO3517) was still predicted incorrectly, demonstrating the previously observed enhanced predictive capabilities of more complete networks [37]

### TIFA essentiality and FBA predictions are in relative close agreement

A direct comparison between TIFA and the previously made direct essentiality calls (DECs) that were based on the presence/absence of insertions in the 80% core sequence of genes [18] is shown in Table 1. Note that FBA gene essentiality predictions for the DEC dataset had to be computed for LB medium instead of SBM. Due to the richer composition of LB, 18 genes fewer were FBA essential, eleven of which could be explained by the presence of tryptophan and pyrimidine in LB (Dataset S1).

**Figure 6:**
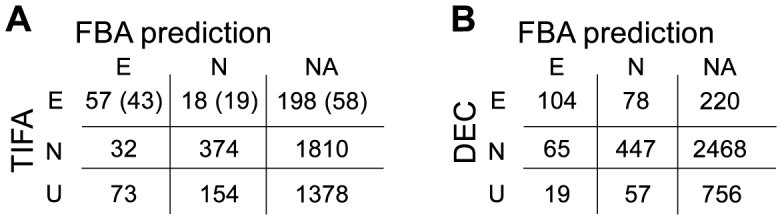
Three-way comparison between FBA predictions and TIFA and DEC essentiality calls. (A) Comparison between FBA and TIFA. (B) Comparison between FBA and direct gene essentiality calls (DEC). The sectors of the comparison matrix show the intersections of FBA essential genes (E), FBA nonessential genes (N) and genes not in the MR-1 model (NA) with TIFA and DEC calls for essential genes (E), nonessential genes (N) and uncalled genes (U). The TIFA essential gene calls that could be alternatively explained by polar operon effects are shown in brackets.

One would expect that the FBA prediction agree more with the TIFA essentiality calls than with the original MR-1 essentiality calls, if 1) TIFA identifications are an improvement over the original direct method, and 2) FBA predicts gene essentiality correctly more often than not. A three-way comparison between the TIFA essentiality calls, DECs, and FBA predictions was used to investigate the relative agreement between the FBA predictions and the two essential gene identification methods. A gene-by-gene comparison is included as supplementary data (Dataset S1). Because all TIFA predictions that could be alternatively explained by polar operon effects had been removed, the number of comparisons between TIFA and FBA were substantially fewer than between the DEC essentiality calls and FBA (Fig. 6). The overall performance of TIFA, expressed as percent correct predictions (combined true essential and true nonessential predictions divided by all essentiality predictions), was much higher than for DEC: 90% vs 79% (Fig. 6), indicating that TIFA calls were indeed better. To investigate the potential influence of polar effects on the essential gene predictions, the TIFA calls that had previously been removed from the dataset because their essentiality could be explained alternatively by polar effects, were compared to the *essential* gene TIFA calls. Note that polar effects are only a problem for essential, and not for nonessential gene calls. The correct prediction percentage of the discarded essential gene predictions was 69% (Fig. 6, 43/(43+19)), which was only slightly lower than the 76% (Fig. 6, 57/(57+18)) for the retained TIFA comparisons. This suggested that polar effects may not result in many false essential gene assignments if these assignments had been used. This was not surprising given that for a polar effect to occur, a downstream neighboring gene in the same operon had been identified as essential.

Genes that were *exclusively* identified as essential by DEC were wrong more often than not (40% correct essential gene predictions, (Dataset S1). Genes exclusively identified as essential using TIFA, had a correct prediction percentage of 68%, which was only slightly lower than the 76% for all TIFA essential gene predictions (Dataset S1). Genes exclusively identified as nonessential by TIFA were in equally good agreement with FBA predictions as the entire TIFA nonessential gene calls (92% compared to 91% agreement). DEC nonessential gene assignments were in 87% agreement with FBA predictions, and exclusive DEC nonessential gene assignments were only 74% in agreement with FBA predictions. In summary, the TIFA gene essentiality calls (both essential and nonessential gene calls) were in much closer agreement with the FBA model predictions than the DEC essentiality calls, providing strong support for TIFA as a better method for gene essentiality calls (Dataset S1).

## Discussion

Tn-seq is a powerful and readily available technology for the genome-wide evaluation of metabolic networks. The observed transposon insertion pattern suggested that 28.6% of essential genes were occasionally inserted, and that insertions in essential genes were not limited to the periphery of genes as was previously presumed [18]. Conversely, several genes that did not contain insertions were *not* identified as essential. Both TIFA, and the previously developed HMM models, are able to identify essential genes containing insertions, but unlike the current HMM models, TIFA explicitly corrects for the observed transposon insertional biases. Note that the confidence associated with an essentiality call was depended on the number of TA locations within a gene. For genes that contained only a handful of TA sites, essentiality could not be established, even if no insertions were found. Hence, to establish essentiality in very short or GC rich genes, a very large mutant library is required. Conversely, genes with many TA locations could be called essential, even if they contained a significant number of insertions. With the current library size, essentiality of 1725 (41%) of the genes could not be called. The observed transposon insertion pattern was in close agreement with Monte Carlo insertion simulations that utilized location specific insertion probabilities. The absence of insertional “hotspots” in comparison to the Monte Carlo simulations was interpreted as validation for the essential gene assignments. The data used for this study was generated from a transposon that was transcriptionally terminated. As a consequence, essentiality could only be determined for a subset of the genes that were identified as essential by TIFA (∼70%, Table 1, 273/(273+120)). A substantial number of genes (120) contained sufficiently few insertions to be identified as essential (Table 1), but the lack of viability could alternatively be explained by the presence of an adjacent downstream essential gene in the same operon. Note that experimental operon predictions from RNA-seq data [38] could improve the here used computational operon projections. Alternatively, utilization of an unterminated read-through transposon would eliminate polar gene essentiality experimentally. The TIFA essential gene predictions agreed fairly well with direct essentiality calls for MR-1[18], but were in closer agreement with essentiality expectations from *E. coli* orthologs (Dataset S1) and FBA predictions of the MR-1 model. In addition, many genes that had been previously identified as essential based on the absence of insertions often contained many insertions in our dataset. TIFA was able to provide transposon insertion-based essentiality calls for 481 of the 770 (62%) non-obsolete genes in the MR-1 model, and was thereby able to perform a comprehensive validation of the MR1 model. For example: the 32 genes incorrectly predicted essential by FBA, suggested that the current MR-1 model was incomplete. And, the 11 TIFA essential genes associated with blocked reactions suggested that some essential reactions in the MR-1 model could not be used, again indicating that the current MR-1 network was incomplete. In addition, FIFA data demonstrated that thermodynamic constraints on the reaction directionalities greatly improved FBA gene essentiality predictions.

## Materials and Methods

### Transposon insertion data

A detailed description of the transposon experiment that generated the data used for this work was published previously [28]. Briefly, a single-mutant library of *S. oneidensis* was generated with the mini*Himar* transposon under kanamycin selection on *Schewanella* Basal Medium (SBM, which is a well-defined rich medium), plates under aerobic conditions [39]. The fitnesses of the pooled clones were evaluated under aerobic conditions using SBM as previously described [4]. Samples of the pooled library were collected before and after a short growth period. The raw sequence data for each sample was mapped to the genome, was filtered to only retain sequences that occurred at least eight times, and that could be mapped uniquely. For TIFA, the data of all four samples were combined to assess the viability of insertional mutants under the condition that were used to generate the library. The fitness values used in the manuscript were calculated from the aerobic treatment data only. Gene fitness was calculated as the median fitness of all clones that were inserted into the same gene (Text S1).

### Estimation of mini*Himar* insertion probabilities

The probability of an insertion occurring *r* times at a given location was approximated by the Poisson distribution: 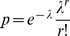, which reduces to 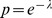 for *r* = 0, where λ is the insertion probability *m/n* with *m* the number of colonies, and *n* the number of TA sites in the genome. This yielded the probability of finding at *least* one insertion for a given location: 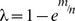, and the total number of observed insertions 
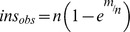
. The number of colonies in the library was estimated by substituting the number of unique sequences in the library 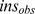, the number of TA sites (*n*) in the genome, and the mean insertion probability *p*. The same approach was followed for determining the sequence-specific insertion probabilities, limiting the analysis to the fraction of TA locations with a given flanking motif (Text S1).

### Identification of essential genes

Assuming equal insertion probability for each TA site, the probability of observing at least the number of experimentally inserted locations in a gene is given by the cumulative probability of the binomial distribution, β(*s*, *p_e_*) which equates to: 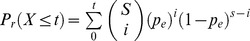, where *p_e_* is the insertion probability for a TA site, *s* is the total number of TA sites in a gene, and *t* the number of observed mutations. A probability generating function was used if equal insertion probability could not be assumed. The general form of *G*(*x*) for each gene was written as: 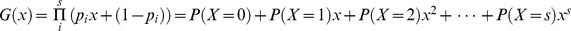, where *s* is the number of TA locations in a gene, and *p_i_* the specific probability for the insertion location. In the power series expansion of *G*(*x*), the coefficient of *x^t^* is the probability *P*(*X* = *t*). The cumulative probability of observing up to *t* insertions in *s* possible TA locations was expressed as 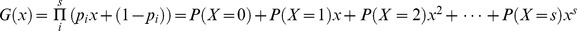. The transposon insertion expectation for a gene was calculated as 
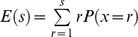
 with a variance of 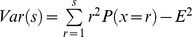. The normalized deviation of expectation (NDE) is given by: 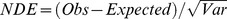.

### FBA essentiality calls

Transposon gene deletion simulations were performed for two different media conditions: LB for the dataset from Deutschbauer [18], and SBM for the dataset from Brutinel [28]. Because both mutant libraries were generated under aerobic conditions, FBA predictions were made for aerobic conditions. To prevent artifacts resulting from unrealisticly large redox exchanges with the media, nutrient uptake *rates* were limited to the *concentrations* in the media (Text S1 for details). The low concentration of metals in both media were therefore unable to sustain dissimilatory metal-reducing growth. Genes were designated FBA essential if removal resulted in <1% biomass production relative to wild type [40] Using zero biomass production as alternative cutoff [41], [42] resulted in identical predictions, suggesting that prediction were insensitive to the cutoff value (Text S1 for details), which was consistent with previous observations for *E. coli* networks [40], [42]. Computationally zero growth was assessed as a biomass production of <1e-6 to eliminate the influence of computational noise.

### Databases and software

The *S. oneidensis* MR-1 metabolic model was previously reconstructed from the original genome annotation [29], which was used in this study for comparison (NCBI, NC_004347.1). Genes that were removed in later annotations (NC 004347.2) were not used. In addition, all TA loci in areas where two genes overlapped were excluded from the dataset. The essentiality of 4,214 genes in the *S. oneidensis* genome was investigated by TIFA. The *S. oneidensis* essentiality and fitness predictions were performed in MATLAB (MathWorks, Natick MA), by using the COBRA toolbox [43] in combination with the linear optimization routine (simplex algorithm) from the CPLEX software suite (IBM, Armonk NY). Operon calls for *S. oneidensis* were taken from ProOpDB [44] and only the terminal genes on operons were used for fitness analysis. Essentiality calls were made for all terminal genes. In addition, upstream genes on the operon were used if they were evaluated as nonessential by using TIFA, or if the directly downstream gene was called nonessential by TIFA. TIFA, genome and Monte Carlo analyses were performed with custom MATLAB and Python (http://www.python.org/) scripts. Unless otherwise indicated, significance was evaluated at *p*<0.05.

## Supporting Information

Dataset S1 **Gene by gene comparison of essentiality, fitness and operon calls.**
(XLSX)

Figure S1 **Properties of essential genes inserted in the gene core 80%.** Of the 273 TIFA identified essential genes, 50 genes contained insertions with associated fitness values. 28 of the 50 contained only insertions in the beginning (10%) and/or in the end (10%), and/or a single insertion in the middle of the gene. Of the remaining 22 genes the insertion location with associated fitness (top diamonds), TA sequences (black diamonds), conserved protein domains (red line segments) and intra gene start codons with associated ribosomal binding site (RBS) strengths (bottom diamonds) are shown. Fitness values outside the visualized range were shown at the extreme ends of the scale. RBS strength was shown logarithmically from −0.03 to 4.3, lower values were omitted. For each gene, the first row underneath the line represents the RBS strengths associated with intra gene start codons of the uninserted gene. Each successive couple of rows represents the RBS strengths of all downstream start codons for each insertion. Two rows are shown for each mutant because the orientation of insertions was unknown. All downstream RBS strengths are shown because a transposon insertion could alter the RBS strength for downstream intra gene start codons. Mutations in three genes (SO4432, SO3185 and SO4669) caused substantially slower growth rates. Insertions in five genes (SO4391, SO0148, SO3873, SO4068 and SO3084) could not be explained from the presented data. For the rest of the genes, insertions were mostly outside conserved protein domains, and the few insertions in conserved domains corresponded to slow growth (SO2545). Insertions in ten genes (SO3178, SO1441, SO3993, SO3874, SO4283, SO2133, SO1442, SO4359, SO0730, and SO2544) may have resulted in a functional protein by reinitiating transcription and translation after transposon insertion.
(PDF)

Text S1 **Additional methods and results.** Supplemental file contains a detailed description of the TIFA method, the Monte Carlo analysis used for the validation of TIFA, and the calculation of fitness values. The supplement contains several additional results.
(PDF)
