# Supplementary figures and images for: Genome-Scale Metabolic Network Validation of Shewanella oneidensis Using Transposon Insertion Frequency Analysis
Source: PLoS Comput Biol. 2014 Sep 18;10(9):e1003848. doi: 10.1371/journal.pcbi.1003848 (PMC4168976; doi:10.1371/journal.pcbi.1003848)

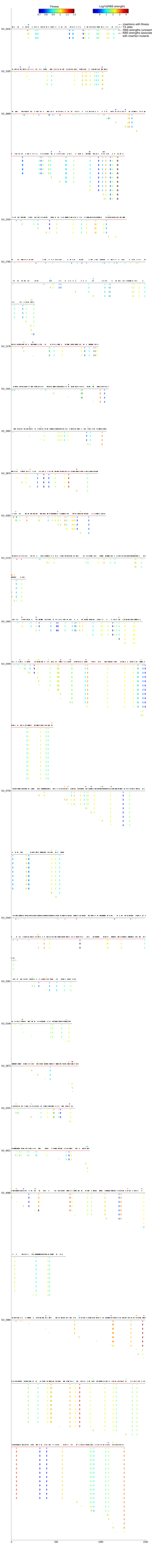

Supplement: Figure S1 — Properties of essential genes inserted in the gene core 80%. Of the 273 TIFA identified essential genes, 50 genes contained insertions with associated fitness values. 28 of the 50 contained only insertions in the beginning (10%) and/or in the end (10%), and/or a single insertion in the middle of the gene. Of the remaining 22 genes the insertion location with associated fitness (top diamonds), TA sequences (black diamonds), conserved protein domains (red line segments) and intra gene start codons with associated ribosomal binding site (RBS) strengths (bottom diamonds) are shown. Fitness values outside the visualized range were shown at the extreme ends of the scale. RBS strength was shown logarithmically from −0.03 to 4.3, lower values were omitted. For each gene, the first row underneath the line represents the RBS strengths associated with intra gene start codons of the uninserted gene. Each successive couple of rows represents the RBS strengths of all downstream start codons for each insertion. Two rows are shown for each mutant because the orientation of insertions was unknown. All downstream RBS strengths are shown because a transposon insertion could alter the RBS strength for downstream intra gene start codons. Mutations in three genes (SO4432, SO3185 and SO4669) caused substantially slower growth rates. Insertions in five genes (SO4391, SO0148, SO3873, SO4068 and SO3084) could not be explained from the presented data. For the rest of the genes, insertions were mostly outside conserved protein domains, and the few insertions in conserved domains corresponded to slow growth (SO2545). Insertions in ten genes (SO3178, SO1441, SO3993, SO3874, SO4283, SO2133, SO1442, SO4359, SO0730, and SO2544) may have resulted in a functional protein by reinitiating transcription and translation after transposon insertion. (PDF) [file pcbi.1003848.s002.pdf]
