# Supplementary material for: Genome-Scale Metabolic Network Validation of Shewanella oneidensis Using Transposon Insertion Frequency Analysis
Source: PLoS Comput Biol. 2014 Sep 18;10(9):e1003848. doi: 10.1371/journal.pcbi.1003848 (PMC4168976; doi:10.1371/journal.pcbi.1003848)
Supplement: Text S1 — Additional methods and results. Supplemental file contains a detailed description of the TIFA method, the Monte Carlo analysis used for the validation of TIFA, and the calculation of fitness values. The supplement contains several additional results. (PDF) [file pcbi.1003848.s003.pdf]

Text S1

Genome-Scale Metabolic Network Validation of *Shewanella oneidensis* using Transposon Insertion Frequency Analysis

Hong Yang, Elias W. Krumholz, Evan D. Brutinel, Nagendra P. Palani1, Michael J. Sadowsky, Andrew M. Odlyzko, Jeffrey A. Gralnick, and Igor G.L. Libourel

Identification of gene essentiality by Transposon Insertion Frequency Analysis (TIFA) 2
Identification of the essential genes 2
Identification of the nonessential genes 5
Fitness calculation 7
Prediction of gene essentiality by Flux Balance Analysis (FBA) 8
References 14

Identification of gene essentiality by Transposon Insertion Frequency Analysis (TIFA)

Identification of the essential genes

First, the cumulative probability value (CPV) was calculated for each gene by assuming that each insertion location has an equal probability. Genes that were likely not essential CPV cutoff of *p>0.1* were selected. Then, the bias of transposon insertion as a function of the insertion location within a gene, the genomic location, and the flanking sequence was determined. The observed biases were corrected for by calculating an insertion probability specific to each TA location in the genome. Only the terminal genes of operons were included in the analysis (2657 genes) to avoid polar insertion effects. Using the corrected specific probability p_{ins} for each insertion, the CPV and normalized deviation of expectation (NDE) were calculated for each gene in the entire gene set. The obtained NDE distribution was evaluated by simulating the transposon insertion experiment using Monte Carlo for the entire gene set, and for the essential genes only. The CPV and NDE distributions were simulated to investigate if insertional hotspots existed. Secondly, the simulation was used to determine the CPV that corresponded to exactly one false positive essential gene assignment (CPV cutoff value). This CPV was nearly identical to the summed marginal essentiality probabilities of the nonessential genes. Genes with the cumulative probability value of less than CPV cutoff were identified as essential genes. The workflow is shown in Fig. S1.

Calculate the insertion probability that a given location is inserted at least once (p_e): By assuming that each insertion location has an equal probability *1/n* (where *n* is the sum of TA locations in the genome), p_e is equal to $1 - e^{-\lambda}$ based on the binomial distribution.

$$p_e = 1 - e^{-\lambda} \ (1)$$

where $\lambda = \frac{m}{n}$, and *m* is the number of insertion events (total colonies).

m can be solved by substitution:

$$n * (1 - e^{-m/n}) = n\_obs \ (2)$$

The left side of Eq. (2) contains the expected number of locations that are inserted, and the right side of the equation contains the total number of unique insertions that were observed from the transposon insertion experiment. p_e may be solved by substituting λ and *m* and combining equations 1 and 2.

Calculate the cumulative probability for each gene assuming equal insertion probabilities: For each gene, the probability of having exactly *t* out of *s* locations inserted *at least once* is given by the binomial distribution $\beta(s, p_e)$. The cumulative distribution function is used to find the probability of observing up to *t* insertions in *s* TA locations:

$$P_r(X \leq t) = \sum_0^t \binom{s}{i} (p_e)^i (1 - p_e)^{s-i} \ (3)$$

Select all genes unlikely to be essential: A CPV cutoff value of $p>0.1$ (data of 15% of all genes were discarded) was used to exclude essential genes for the investigation of bias associated with the position of insertions in the genome or the nucleotide sequence flanking the inserted TA positions.

Evaluate the bias of transposon insertions: First, the insertion location bias within genes was investigated. Each gene was divided into 0.2% windows. The ratio of the inserted TA to the total number of TA locations was plotted. No insertion distribution bias was observed. Only genes unlikely to be essential cutoff value of ($p>0.1$) were used to evaluate if transposon insertions were dependent on their genomic location or flanking sequence. To investigate if insertion probabilities were a function of genome location, the number of insertions per TA site was plotted as a function of the location on the genome. To calculate the average number of insertions per TA site, we chose a window size of 20,000 base pairs, and moved this window with 2,000 base pairs increments. For each window, the probability that a given TA location was inserted at least once was calculated from the binomial distribution model as before. The equation that best described the observed pattern was a combination of a quadratic and an absolute linear equation (Eq. (4)). The coefficients in the equation were estimated using a least-square method.

$$p_{loc} = ax^2 + b|x| + c \quad (4)$$

Next, the influence of the two upstream and two downstream nucleotides surrounding insertion sites was investigated. Each of the four considered nucleotide positions has four outcomes: (ATCG). Therefore, a solution space of 4*4*4*4 = 256 independent outcomes existed. Of these 256 combinations, 120 sequences are complementary to each other, and 16 sequences are palindromic sequences (e.g. AATATT). Therefore, there are 136 unique outcomes. For each outcome, the total number of occasions in the genome and the number of inserted occasions from the transposon insertion experiment was determined. A p_{seq} was calculated for each of the 136 motifs using the binomial distribution model.

Calculate the bias-corrected insertion probability for each TA location: The probability that a given location is inserted at least once was calculated by multiplying its sequence specific probability by its scaled location probability, which is equal to its location probability divided by average location probability.

$$p_{ins} = p_{seq} * p_{scaled-loc} \quad (5)$$

Calculate the normalized deviation of expectation (NDE) and recalculate the cumulative probability value (CPV) based on the location specific probability: For each gene, the probability of having exactly *t* out of *s* TA locations inserted at least once is not given by a binomial distribution anymore because each insertion location has a different insertion probability. Therefore, we used a probability generating function.

The general form of $G(x)$ for each gene can be written as $G(x) = \prod_i^s (p_i\, x + (1 - p_i)) = P(X = 0) + P(X = 1)\, x + P(X = 2)\, x^2 + \cdots + P(X = s)\, x^s$, where *s* is the total number of TA sites in a gene, and p_i is the specific probability for the insertion probability of location *i*. In the power series expansion of $G(x)$, the coefficient of x^t is the probability $P(X = t)$. So the cumulative probability value, the probability of observing up to *t* insertions in *s* possible insertion locations, can be expressed as$P(X \le t) = \sum_{r=0}^{t} P(x = r)$. The expected number of insertions for each gene can be calculated as $E(s) = \sum_{r=1}^{s} rP(x = r)$ and the variance is calculated as $Var(s) = \sum_{r=1}^{s} r^2\, P(x = r) - E^2$. Then, the normalized deviation of expectation (NDE) for each gene can be calculated as $NDE = (Observed - E)/\sqrt{Var}$.

For example, there are 2 insertions observed in a gene that has 5 TA locations. The specific probability for these 5 insertion locations were calculated as following: for example 0.1, 0.3, 0.2, 0.5, and 0.4. Therefore, $G(x) = (0.1 + 0.9x) * (0.3 + 0.7x) * (0.2 + 0.8x) * (0.5 + 0.5x) * (0.4 + 0.6x) = \frac{3}{2500} + \frac{107}{5000}x + \frac{637}{5000}x^2 + \frac{1637}{5000}x^3 + \frac{1857}{5000}x^4 + \frac{189}{1250}x^5$. The cumulative probability value of observing up to two insertions is equal to $\mathrm{P}(X \le 2) = \sum_{r=0}^{2} P(x = r) = \mathrm{P}(x = 0) + (x = 1) + (x = 2) = \frac{3}{2500} + \frac{107}{5000} + \frac{637}{5000} = 0.15$. The expected number of insertion is equal to $E(5) = \sum_{r=1}^{5} rP(x = r) = 1 * \frac{107}{5000} + 2 * \frac{637}{5000} + 3 * \frac{1637}{5000} + 4 * \frac{1857}{5000} + 5 * \frac{189}{1250} = 3.5$, and the variance of insertion is equal to $Var(5) = \sum_{r=1}^{5} r^2\, P(x = r) - E^2 = 1^2 * \frac{107}{5000} + 2^2 * \frac{637}{5000} + 3^2 * \frac{1637}{5000} + 4^2 * \frac{1857}{5000} + 5^2 * \frac{189}{1250} - 3.5^2 = 0.95$. The NDE is then equal to $NDE = \frac{Observed - E}{\sqrt{Var}} = \frac{3 - 3.5}{\sqrt{0.95}} = -0.5130$.

Simulate the transposon insertion experiment using Monte Carlo simulation: We generated a random number for each TA insertion location in the genome. If the generated number was less than the specific insertion probability (p_{ins}) for that site, the insertion event was counted as successful, and the number in the simulated matrix was set to 1. Otherwise, the number in the simulated matrix was set to 0. For each insertion location, the simulation procedure was repeated 1000 times. This Monte Carlo simulation generated a matrix of simulated insertion with *n* rows (total TA locations in the genome) and 1000 columns (number of simulations), with each element binary.

Calculate the normalized deviation of expectation (NDE) and the cumulative probability value (CPV) for the simulated experiment: The NDE and CPV for the simulated data were calculated the same as for the experimental data. This process generated two matrixes: one contained the CPV for each gene and 1000 columns representing each Monte Carlo run, and the second contained the NDE for each gene and 1000 columns representing each Monte Carlo run.

Compare the simulated data with the experimental data: To validate essential gene identification through transposon insertion frequency analysis (TIFA), the simulated data was

compared to the observed insertion data. The absence of groups of genes with more insertions than expected would suggest that the formulated probability model is an accurate description of the insertion behavior of the mariner transposon. We therefore investigated whether genes existed in the genome with a higher number of insertions than expected from our insertion probability model using NDE. The data was grouped with a bin width of 0.5. The number of genes falling into each bin was calculated for the experimental data, and for the simulated data, with the average and standard deviation of the 1000 simulations. Only one bin of 3 genes had a much higher experimental NDE value than was simulated, suggesting that insertional hotspots were very rare or absent.

Choose the cutoff CPV for gene essentiality: The CPV cutoff value was calibrated by progressively increasing its value until exactly one gene of the Monte Carlo simulation falsely identified as essential. The same CPV was found by summing the marginal probabilities of all nonessential genes to be found essential by chance.

Identify the essential genes using the CPV cutoff: All genes with a lower cumulative probability value than the CPV cutoff value were identified as essential. However, genes for which the essentiality score could be explained alternatively by their relative position in an operon to another essential gene (polar essentiality) were not identified as essential, but were designated as “unknown” instead.

Identification of the nonessential genes

Essential genes were identified by containing significantly fewer insertions than expected from a nonessential gene transposon insertion model. To identify *nonessential* genes, an *essential* gene transposon insertion model was formulated. The average insertion frequency for essential genes was calculated by dividing the number of experimentally observed insertions by the expected number of insertions for the group of genes that fell outside the expected insertion distribution as determined by the Monte Carlo simulation. Note that this group was larger than the group of genes that could be identified as essential. This ratio was multiplied by the specific probability for each TA location to create an essential gene probability model. The cumulative probability value for each gene was calculated for the essential gene model, and nonessential genes were identified by containing significantly more insertions than expected, again using a one-gene false positive cutoff value for the CPV, which was determined as before. Genes that could not be identified as essential or nonessential were labeled as unknown.

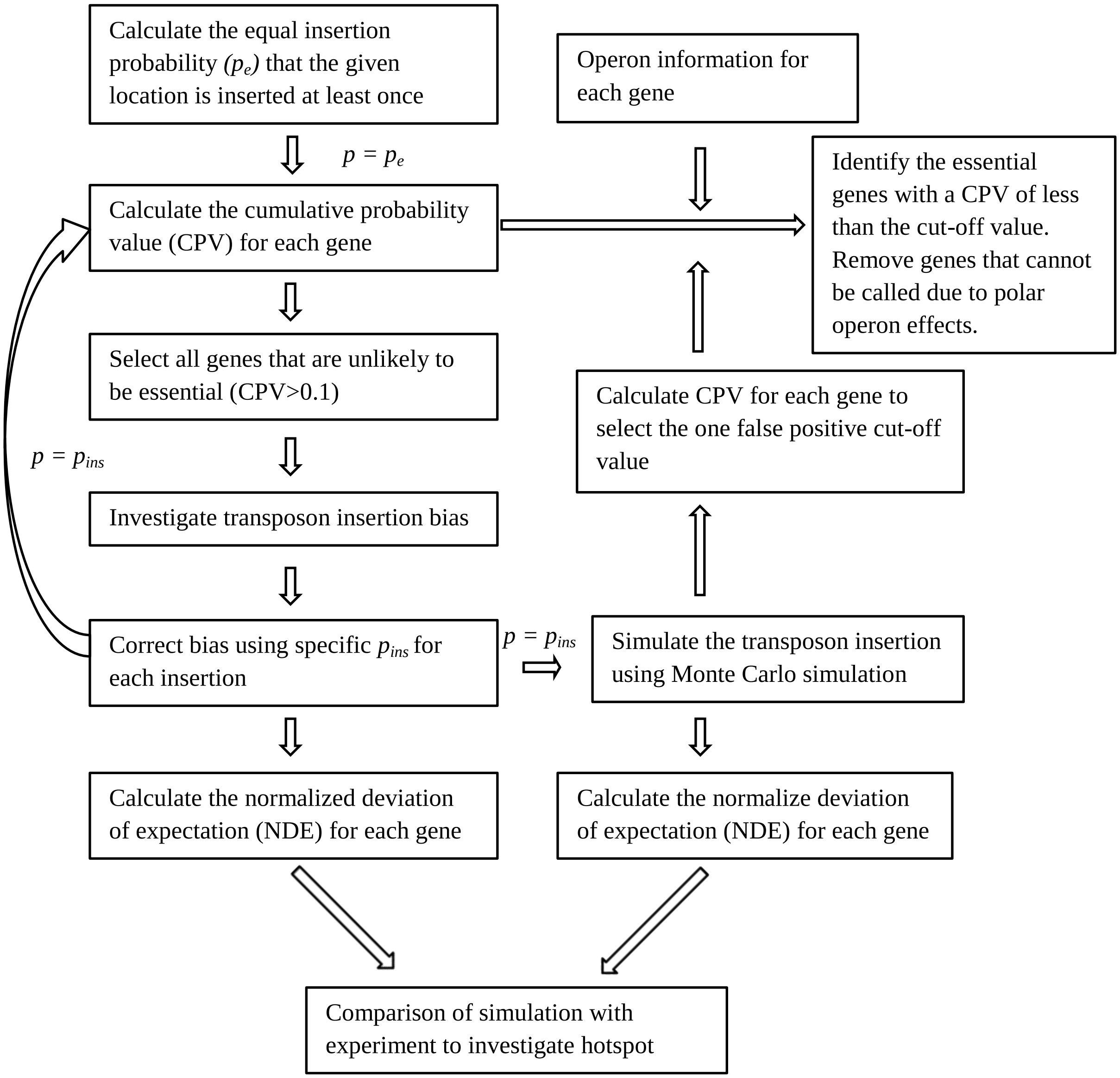

Fig. S1: Workflow for the identification and quantification of gene essentiality by TIFA.

Fitness calculation

Fitness, used here as a proxy for growth rate, was calculated as by Opijnen et al. (2009) [4]. First, the fitness value for each insertion location was calculated by comparing the fold expansio1n of the mutant relative to the rest of the population with the following equation:

$$W_i = \frac{\ln[N_i(t_2) * \frac{d}{N_i(t_1)}]}{\ln[\left(1 - N_i(t_2)\right) * \frac{d}{1 - N_i(t_1)}]}$$

where $N_i(t_1)$ and $N_i(t_2)$ are the frequency of a mutant in the population (number of reads of a specific location normalized by the total number of reads per time point) at the start and the end of the experiment, respectively. d (expansion factor) is the population size at t_1 (OD_{600} of 0.005) relative to t_2 (OD_{600} of 0.2). Fitness values were only calculated for the observed insertions with a number of reads of more than 8 for both time points.

The median, mean and standard deviation of fitness values for each gene were calculated by including all observed insertions in a gene.

Prediction of gene essentiality by Flux Balance Analysis (FBA)

FBA was used to infer the potential of knockout strains to produce biomass. Simulated knockout strains that were able to produce biomass were expected to be viable, and the eliminated gene was referred to as FBA-nonessential.

$$max\ v_{biomass}$$

$$s.t. \sum_{j=1}^{M} S_{ij} v_j = 0, i = 1, 2, \dots, N$$

$$v_j^{min} \le v_j \le v_j^{max}, j = 1,2, \dots, N$$

where *M* is the number of fluxes, *N* is the number of metabolites, *S* is the stoichiometric matrix of the metabolic network, $v_{biomass,max}$ is the maximal value for the biomass production, v_j^{min} and v_j^{max} are the lower and upper bound for each flux *j*, respectively. For knockout strains the constraint $v_j = 0, j \in G$ is added that forces zero flux through the reaction(s) associated with a deleted gene.

An *S. oneidensis* MR-1 model was previously reconstructed and published [29]. The MR-1model includes 774 reactions, 783 genes and 634 unique metabolites. A biomass objective function was formulated based on the experimental measurements for *S. oneidensis* MR-1 grown on lactate under aerobic conditions. For wild-type and each single gene knockout, biomass production was assessed using FBA allowing the uptake of all metabolites known to be present in the supplied medium and for which exchange reactions between the media and cell existed in the model [28]. The lower bounds of exchange reactions were adjusted to the concentrations of metabolites in the medium (Table A). Metal ions were supplied in equal amounts for all oxidation states: for example Fe^{2+} and Fe^{3+} were set to the same concentration. The lower bounds of O_2, H^+, H_2O, CO_2 were set to -1000 allowing free exchange. All the upper bounds of exchange reactions were set to 1000

Table A: SBM and LB composition used to limit FBA nutrient uptake

Metabolite	SBM (mM)	LB (mM)	FBA
H_2O	1000	1000	+
O_2	1000	1000	+
H^+	1000	1000	+
Phosphate	2.97	4.44	+
Sulfate	2.63	0.39	+
Cl^-	8.30	171.82	+
K^+	4.25	4.92	+
Na^+	208.47	189.19	+
Mg^{2+}	0.91	0.23	+
Ammonia	3.41	-	+
L-Lactate	20	-	+
D-Lactate	20	-	+

Fumarate	80	-	+
L-Alanine	0.03367	6.73476	+
L-Arginine	0.01435	2.29621	+
L-Aspartate	0.03757	5.89782	+
L-Cysteine	0	0	+
L-Cystine*	0.00042	0.16646	-
L-Glutamate	0.10807	13.45749	+
L- Glutamine	0	0.10264	+
L-Glycine	0.01865	4.26269	+
L-Histidine	0.01225	1.64357	-
L-Isoleucine	0.03049	5.33659	+
L-Leucine	0.03812	7.28063	+
L-Lysine	0.04036	5.81435	+
L-Methionine	0.00938	1.67549	-
L-Phenylalanine	0.02179	3.93486	-
L-Proline	0.06949	6.60123	-
L-Serine	0.01903	2.85470	+
L-Threonine	0.01427	2.18267	+
L-Tryptophan	0	0.51414	+
L-Tyrosine	0.00221	1.04862	+
L-Valine	0.04780	6.53009	+
Cu^{2+}	0.00080	0.00561	+
Ca^{2+}	0.00015	0.08009	+
Co^{2+}	0.00357	0.00297	+
Cd^{2+}	-	6.672e-5	-
Fe^{2+}	0.00540	0.01343	+
Fe^{3+}	0.00540	0.01343	+
Hg^{2+}	-	NA	+
Mn^{2+}	0.00253	0.00021	+
Zn^{2+}	0.00367	NA	-
Arsenate	-	NA	+
Chromate	-	0.00115	+
MoO_4^{2-}	0.00219	-	+
Ni^{2+}	0.00463	-	+
WO_4^{2-}	0.00030	-	+
SeO_4^{2-}	0.00265	-	+
Folate	2.26552e-5	0.00023	-
Lipoate	0.00012	NA	-
Niacin	0.00020	0.01218	-
PAN	0.00010	0.00205	-
Pyridoxal (B6)	0.00024	0.00071	-
Riboflavina	6.64258e-5	0.00066	-
Thiamine Phosphate (B1)	7.41246e-5	0.00030	+
Vitamin B12	3.72476e-7	1.862e-8	+
AMP	-	0.54631	-
CMP	-	0.36417	-
GMP	-	0.32404	-
UMP	-	0.58513	-
Adenosine	-	4.323e-6	+
Guanosine	-	6.650e-5	-
Inosine	-	0.00103	+
Thymidine	-	NA	+
Uridine	-	0.06949	+
Uracil	-	0.03368	+
Deoxyadenosine	-	9.975e-5	+
Deoxycytidine	-	NA	+
HYXN (Hypoxanthine)	-	0.00921	-
D-glucose	-	0.00105	-
H_2S	-	NA	
Heme	-	NA	-
Prephenate*	-	NA	-
Shikimate*	-	0.00047	-

The lower bounds for FBA analysis were set to the concentrations of media compounds in SB and LB media. '*' Compounds in LB media but not in Argonne LB [22]. '-' Metabolites not present in media. 'NA' metabolites in LB media definitions, but the concentrations were not available. Bounds for NA metabolites were set to zero. MR-1 model contained '+', or did not contain '-' exchange reaction.

Source and description of contents of media: The SBM medium consisted of (per liter) 0.225g K_2HPO_4, 0.225g KH_2PO_4, 0.46g NaCl, 0.225g $(NH_4)_2SO_4$, 0.117 $MgSO_4{\cdot}7H_2O$, and 10 mM (aerobic) or 100 mM (anaerobic) HEPES, adjusted to pH 7.2 [28]. In addition, 5ml l^{-1} trace mineral mix (containing per liter: 1.5g NTA, 0.1g $MnCl_2{\cdot}4H_2O$, 0.3g $FeSO_4{\cdot}7H_2O$, 0.17g $CoCl_2{\cdot}6H_2O$, 0.1g $ZnCl_2$, 0.04g $CuSO_4{\cdot}5H_2O$, 0.005g $AlK(SO_4)_2{\cdot}12H_2O$, 0.005 H_3BO_3, 0.09g Na_2MoO_4, 0.12g $NiCl_2$, 0.02g $NaWO_4{\cdot}2H_2O$, and 0.10g Na_2SeO_4) [45], 5 ml l^{-1} of Wolfe's vitamin solution excluding riboflavin (per liter: 2mg biotin, 2mg folic acid, 10mg pyridoxine hydrochloride, 5mg thiamine hydrochloride, 5mg nicotinic acid, 5mg DL-calcium pantothenate, 0.1mg vitamin B12, 5mg *p*-aminobenzoic acid and 5mg lipoic acid) [46], 0.01% casamino acid (Bacto), 40mM sodium DL-lactate (ratio of D isomer to L isomer of 1:1) and 80mM fumarate were added.

LB medium was approximated by (per liter) 10g tryptone, 5g yeast extract and 10g NaCl. Concentrations of amino acids, Ca^{2+}, Mg^{2+}, K^{+}, Na^{+}, Cl^{-},sulfate and phosphate contained in tryptone and yeast extract were calculated from the analysis reports of BD tryptone Bacto™ and yeast extract™ (http://www.bd.com/resource.aspx?IDX=9572). Free amino acid levels were used in place of total amino acid levels if no total values were available. Concentrations of trace elements including Cd, Co, Cr, Cu, Fe, Mg, Mn, Mo, and Ni were calculated according to the their average values of yeast extract [47]. Concentrations of vitamins in yeast extract were calculated based on Sigma-Aldrich product report except vitamin B_{12} reported by National Nutrient Database in USDA (http://ndb.nal.usda.gov/ndb/foods/show/8407?qlookup=yeast+extract&fg=&format=&man=&lfacet=&max=25&new=1). Concentrations of ribonucleotides were calculated from the RNA/protein ratio in yeast extract [48] and GC content of *Saccharomyces cerevisiae*. Concentration of the nucleosides and nucleobases were approximated with the intracellular concentrations in *E.coli* normalized to the intracellular glutamate concentration and glutamate weight in yeast extract [49]. Glucose concentration was taken from a measurement using glucose-binding protein (GBP) for *E. coli* [50].

Sensitivity of FBA mutant predictions to biomass production criterion: The FBA gene essentiality predictions were based on biomass production after gene deletion. In this study, we chose a <1% wild-type biomass production as cutoff for FBA essentiality predictions, consistent with previous work [40]. To investigate sensitivity to the chosen cutoff, the mutants were sorted by ascending biomass production (Fig S2). For SBM, one gene (*amtB*) switched to essential when the cutoff value was set as 10% of wild-type biomass. A cutoff of 50% of wild-type biomass production resulted in an additional nine genes (*argF*, *argG*, *argH*, *leuA*, *leuB*, *leuC*, *leuD*, *ilvC*, *ilvD*) that were predicted FBA-essential. The MR-1 FBA predictions for LB medium were more sensitive to the percent biomass cutoff (Fig. S2).

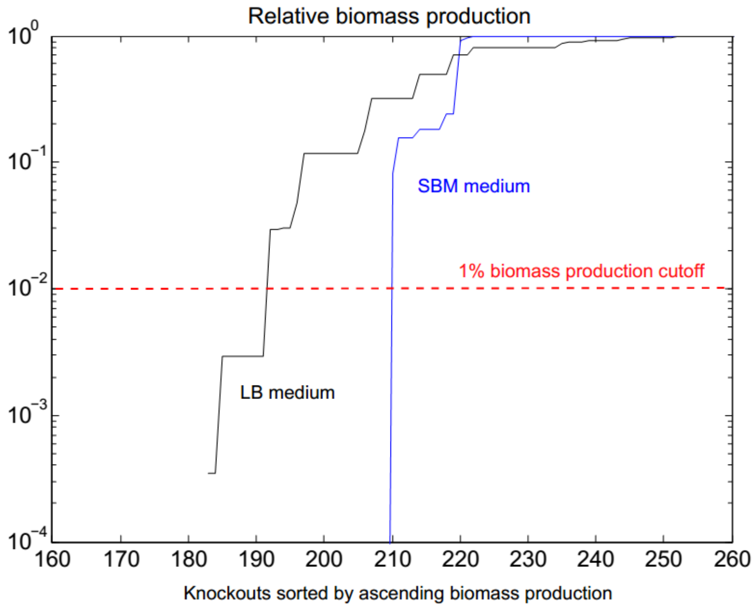

Fig. S2: Mutant with predicted intermediate biomass production

LB and SBM compounds for which no reported concentrations could be found in the literature were assumed zero. To investigate the modeling consequences of no uptake of these metabolites, the FBA results were compared to outcomes of unbounded uptake. SO1335 (thymidylate synthase) became nonessential if thymidine uptake was unlimited. No other zero concentration metabolite bound influenced essentiality predictions. SO1335 was identified as essential by DEC and not called by TIFA, suggesting that zero bounds did not influence comparisons did positively influence DEC outcomes, and did not influence TIFA outcomes.

Comparison of essential genes

The 57 correctly predicted aerobic essential genes were involved in glycolysis/gluconeogenesis (3); amino acid metabolism (23); fatty acid synthesis (2); protein synthesis (1); nucleotide salvage pathways (1); cofactor and prosthetic group biosynthesis (6); purine and pyrimidine biosynthesis (8); cell envelope biosynthesis (11); the alternative carbon pathway (1), and an unassigned gene. This left about 24% transposon insertion identified as essential genes that were predicted to be nonessential by FBA (18 genes, false negative predictions). These genes were involved in glycolysis/gluconeogenesis (1); amino acid metabolism (3); citrate acid cycle (2); cofactor and prosthetic group biosynthesis (10); cell envelope biosynthesis (1); and folate metabolism (1) (Table B).

Table B: False negative model prediction for gene essentiality

Scenario	Metabolism	Genes
Associated with the blocked reactions (11)	Folate metabolism<br>Cofactor and prosthetic group biosynthesis	SO0031 (*fmt*)<br>SO4314 (*hemD*), SO0435 (*hemE*), SO0468 (*ubiA*), SO2741 (*bioAec*), SO2740 (*bioBec*),SO1525 (*dxs*), SO3108 (*sirf*), SO3529 (*lytB*), SO3638 (*pdxA*), SO3653 (*ispB*)
Others (7)	Cell envelope biosynthesis<br>Citrate cycle<br>Glycolysis/Gluconeogenesis<br>Amino acid metabolism	SO4274 (*bacA1*)<br>SO0432 (*acnB*) ,, SO1931 (*sucB*)<br>SO2644 (*ppsa*)<br>SO0862 (*serA*),SO3413 (*thrA*), SO2767 (*asnB*)

Comparison of nonessential genes

Of the 2216 TIFA identified nonessential genes, 374 genes were predicted to be nonessential and 32 genes were predicted to be essential by the FBA. 374 correctly predicted aerobic nonessential genes from the model were involved in glycolysis/gluconeogenesis (6); amino acid metabolism (63); citric acid cycle (9); fatty acid synthesis (4); pentose phosphate pathway (5); pyruvate metabolism (4); folate metabolism (4); nucleotide salvage pathways (16); cofactor and prosthetic group biosynthesis (36); purine and pyrimidine biosynthesis (4); cell envelope biosynthesis (13); energy metabolism (64); alternative carbon pathway (26); transporters (101); and 19 other. This left about 7.88 % TIFA identified as nonessential that were predicted to be essential by FBA (32 genes, false positive prediction). These genes were involved in glycolysis/gluconeogenesis (3); amino acid metabolism (11); cofactor and prosthetic group biosynthesis (4); purine and pyrimidine biosynthesis (2); cell envelope biosynthesis (5); folate metabolism (1); pyruvate metabolism (2); fatty acid synthesis (1); alternative carbon pathway (2) and unassigned (1) (Table C).

Table C: False positive FBA essentiality predictions

Scenario	Metabolism	Genes
Biomass component (2)	Glycogen<br>LPS	SO1498 (*glgC*)<br>SO3745 (*rfaEec*)
Others (30)	Pyruvate metabolism<br>Glycolysis/Gluconeogenesis<br>Amino acid metabolism<br><br><br><br>Cofactor and prosthetic group biosynthesis<br>Purine and pyrimidine biosynthesis<br>Cell envelope biosynthesis<br><br>Folate metabolism<br>alternative carbon pathway<br>Unassigned	SO2912(*pflA*), SO2913(*pflB*)<br>SO3547(*pgi*), SO1499 (glgA)<br>SO0040 (*aroE*), SO1367(*pheA*), SO4056 (*metB*), SO2072(*hisC*), SO2073 (*hisD*), SO1871(*speD*), SO3763 (*speE*), - SO2341 (*bkdC*), - SO1676 (metA), SO2068 (hisF),<br>SO0613 (pabA),<br>SO2342 (*nadA*), SO1341(*nadB*), SO3468(*ribE*), SO2296 (ribEH3)<br>SO1301(*pryB*), SO4255(*pyrE*),<br>SO0194 , SO2088(*msbB*), SO3189(*wbpP*), SO4054(*metF*), SO0567 (*plsC*)<br>SO2774(*fabF*)<br>SO1665(*galU*), SO2336(*pgm*)<br>SO2474(carbonic anhydrase family protein)

References

45. Marsili E, Baron DB, Shikhare ID, Coursolle D, Gralnick JA, et al. (2008) *Shewanella* secretes flavins that mediate extracellular electron transfer. Proc Natl Acad Sci U S A 105: 3968-3973.
46. Balch WE, Fox GE, Magrum LJ, Woese CR, Wolfe RS (1979) Methanogens: reevaluation of a unique biological group. Microbiol Rev 43: 260-296.
47. Grant CL, Pramer D (1962) Minor element composition of yeast extract. J Bacteriol 84: 869-870.
48. Yamada EA, Sgarbieri VC (2005) Yeast (*Saccharomyces cerevisiae*) protein concentrate: preparation, chemical composition, and nutritional and functional properties. J Agric Food Chem 53: 3931-3936.
49. Guo AC, Jewison T, Wilson M, Liu Y, Knox C, et al. (2013) ECMDB: the *E. coli* Metabolome Database. Nucleic Acids Res 41: D625-630.
50. Ge XD, Tolosa L, Simpson J, Rao G (2003) Genetically engineered binding proteins as biosensors for fermentation and cell culture. Biotech and Bioeng 84: 723-731.
